# Inversion of Rayleigh Wave Dispersion Curves via Long Short-Term Memory Combined with Particle Swarm Optimization

**DOI:** 10.1155/2022/2640929

**Published:** 2022-12-23

**Authors:** Yu Fu, Angen Yang, Zhenan Yao, Yuchen Liu, Hongxing Li, Haonan Chen, Xiangteng Wang

**Affiliations:** ^1^Engineering Research Center for Seismic Disaster Prevention and Engineering Geological Disaster Detection of Jiangxi Province (East China University of Technology), Nanchang 330013, China; ^2^Qingdao National Laboratory for Marine Science and Technology Development Center, Qingdao 266237, Shandong Providence, China

## Abstract

An essential step in surface wave exploration is the inversion of dispersion curves. By inverting dispersion curves, we can effectively establish the shear-wave velocity model and obtain reliable subsurface stratigraphic information. The inversion of dispersion curves is an inversion problem with multiple parameters and multiple poles, and obtaining a high precision solution is difficult. Among the methods of inversion of dispersion curves, local search methods are prone to fall into local extremes, and global search methods such as particle swarm optimization (PSO) and genetic algorithm (GA) present the disadvantages of slow convergence speed and low precision. Deep learning models with strong nonlinear mapping capability can effectively solve nonlinear problems. Therefore, we propose a method called PSO-optimized long short-term memory (LSTM) network (PSO-LSTM) to invert the dispersion curves in order to improve the effect of inversion of dispersion curves. The method is based on the LSTM network, and PSO is used to optimize the LSTM network structure and other parameters that need to be given manually to improve the prediction of the network. Two theoretical geological models are used in the paper: Model *A* and Model *B* to test the PSO-LSTM. The tests include the noisy data test and noise-free data test. Model *A* was tested without noise, and Model *B* was tested with noise. In addition, PSO and LSTM were tested on model *A* to compare the performance of PSO-LSTM. In Model *A*, the maximum relative errors of PSO and LSTM are 20.76% and 5.85%, respectively, and the maximum standard deviations of PSO and LSTM are 57.37 and 1.97, respectively. For PSO-LSTM, the maximum relative errors of Model *A* and Model *B* in the inverse results are 2.05% and 2.09%, and the maximum standard deviations of Model *A* and Model *B* in the inverse results are 1.23 and 3.87, respectively. The test results of Model *A* show that the inversion performance of PSO-LSTM is better than those of LSTM and PSO, and the performance of the network can be improved after PSO is used to optimize the network parameters. The inverse results from Model *B* show that the PSO-LSTM is robust and can invert the dispersion curves well even after adding noise to the model. Finally, the PSO-LSTM is used to invert the actual data from Wyoming, USA, which demonstrates that the PSO-LSTM can be used for the quantitative interpretation of Rayleigh wave dispersion curves.

## 1. Introduction

Rayleigh waves are waves that transmit along the surface or medium partition interface formed by the interference and superposition of *P*-wave and *S*-waves. Since the discovery of Rayleigh waves by the British scholar Rayleigh [1], scholars have been investigating theories related to the propagation of Rayleigh waves in the strata. In the early stages of research, Rayleigh waves were treated as noise, and researchers focused on the characteristics of Rayleigh waves to reduce their hazard in earthquakes or to eliminate their impact on valid information in oil seismic exploration [2]. With the deepening of research, scholars found that Rayleigh waves propagate in layered media with dispersion phenomenon. Scholars then began to use Rayleigh waves in the study of stratigraphic structure. Compared with the conventional exploration methods, such as reflection seismic exploration, surface wave exploration has the advantages of nondestructive testing, convenient construction, high resolution at shallow depth and fast detection speed [3] and is broadly used in the fields of soil delineation, engineering nondestructive testing, and geological hazard survey.

Surface wave exploration can be divided into three steps: surface wave data acquisition, dispersion curves picking, and inversion of dispersion curves. As the key step of surface wave exploration, the inversion of dispersion curves directly affect the reliability of the requested stratigraphic information. Currently, there are two main types of methods to invert the dispersion curves: local search methods and global search methods. Local search methods include the least squares method, Levenberg–Marquardt (L-M) algorithm, and Occam algorithm. Dorman and Ewing [4] used the damped least squares method to invert the dispersion curves for the first time. The *S*-wave velocities obtained by the inversion were consistent with those obtained by the refracted wave method, confirming the validity of the method. Xia et al. [5] combined the L-M algorithm and the singular value decomposition technique to invert the Rayleigh wave phase velocity. The method improved the convergence speed, computational performance, and stability of the inversion. In addition to least squares method, the Occam algorithm has also been applied to the study of inversion of dispersion curves. For example, Ai and Cheng [6] employed the Occam algorithm for the inversion of Rayleigh wave dispersion curves, and the results of inversion showed that the Occam algorithm can balance the accuracy of model and the computational rate of the inversion well. Local search methods are extensively used because of their rapid computational speed. However, such methods rely excessively on the initial model, and reliable results of inversion can be obtained only if the initial model is similar to the real model. In addition, local search methods are prone to fall into local extremes, and the partial derivatives involved in the calculation and the results of inversion are affected by the accuracy of the Jacobi matrix. The development of local search methods is limited by these factors. Therefore, researchers have applied another global research method that can avoid the initial model selection and partial derivative calculation to invert dispersion curves, such as GA and simulated annealing algorithm (SA). Shi and Jin [7] used GA for inversion of dispersion curves. In the inversion, Shi and Jin modified the search range by analyzing the initial search results, and thus improved the search efficiency of the method. Yamanaka and Ishida [8] added an elite screening strategy to GA to promote the convergence of the solution. Dal Moro et al. [9] used GA and a posteriori probability density estimation for inversion of dispersion curves, and the method obtained solutions with higher accuracy compared to those obtained by GA. In addition, some scholars have also used SA for inversion of dispersion curves studies. Lu et al. [10] proposed a heat-bath simulated annealing algorithm based on the SA. The inverse results showed that the heat-bath simulated annealing algorithm is more suitable for inversion of dispersion curves than the L-M algorithm. Compared with local search methods, the above global search methods are superior, but these global search methods with long operation time and low solution accuracy are still needed to improve for high precision surface wave exploration.

Deep learning models are capable of building a good mapping between signal and semantics by building a hierarchical structure similar to the human brain, extracting features layer by layer from the bottom to the top of the input data [11]. Deep learning models are excellent at solving nonlinear problems and making fast predictions, and their applications in the geophysical field are gradually increasing in recent years, such as earthquake detection and localization [12], seismic lithology prediction [13], denoising [14], detecting faults [15], and other directions. Deep learning techniques have also yielded impressive results in the field of surface wave exploration. Dai et al. [16] proposed a network specifically for dispersion curve extraction called dispersion curves network (DCNet). The network can extract the dispersion curves quickly and accurately. Theoretical and practical data test results show that the accuracy of the extracted dispersion curves using DCNet has reached the level of manual pickup and can meet the needs of practical work. Song et al. [17] proposed a neural network Res-Unet++, which can accurately and efficiently extract the dispersion curves. Actual data have verified that using this network to select the dispersion curves is better than that of manual selection. Yablokov et al. [18] developed an artificial neural network for Rayleigh surface wave fundamental mode dispersion curve inversion. The accuracy of the inverse results of this method is better than the Monte Carlo algorithm inverse results and similar to the Gray Wolf optimization inverse results by theoretical model testing. For noisy data, the artificial neural network still works well. Wu et al. [19] proposed a LSTM network to invert surface wave based on the first height last velocity loss function. The test results of synthetic and real data show that the network can be effectively used not only for theoretical data inversion but also cope well with real data. The results of dispersion curves inversion mentioned above were summarized as shown in Table 1.

On this basis, the PSO-LSTM is used in this paper for the study of inversion of dispersion curves. PSO is used in the selection of parameters of the LSTM for the number of neurons in the hidden layer, the learning rate, and the number of training rounds in the LSTM to avoid the low prediction accuracy of the network model due to improper manual tuning of the parameters. In order to evaluate the ability of the PSO-LSTM to invert dispersion curves in detail, the feasibility of the model for inversion of dispersion curves was first verified by using a model without noise; then the stability of the PSO-LSTM was tested by using a model with 10% Gaussian noise; and finally, the ability of the PSO-LSTM to invert the actual data was tested by using seismic data from the Wyoming area.

## 2. Principle and Implementation of PSO-LSTM

### 2.1. Long and Short-Term Memory Network

**Figure 1:**
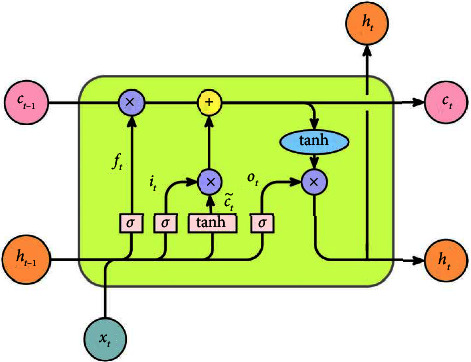
LSTM basic cell structure [20, 21].

The LSTM introduces a gating mechanism, which is better at handling timing problems than the traditional recurrent neural network. The cell structure of the LSTM is shown in Figure 1. In Figure 1, *f*_*t*_, *i*_*t*_, and *o*_*t*_ denote the forget gate, input gate, and output gate, respectively, *c*_*t*_ denotes the neural unit state, *h*_*t*_ denotes the hidden layer state, *x*_*t*_ denotes the input vector of the LSTM unit, *σ* and tanh denote the sigmoid and tanh activation functions, respectively.

The core components of the LSTM are the cell state and the gate structure; the cell state can be seen as a channel for information transfer, allowing information to be passed continuously; the gate structure continuously learns during the training process whether to keep or forget information. The input gate determines the important information in the current input, which in turn updates the cell state; the forget gate determines the information that should be discarded or retained; the output gate is used to determine the new hidden *h*_*t*_, and to pass the new cell state *c*_*t*_ and the new hidden state *h*_*t*_ to the next LSTM cell. The information transfer within the LSTM neural unit follows equation (1)–(6):

(1) $$\begin{array}{c} f_{t}=\sigma \left(W_{f}\cdot \left[h_{t-1},x_{t}\right]+b_{f}\right)\text{,} \end{array}$$

(2) $$\begin{array}{c} i_{t}=\sigma \left(W_{i}\cdot \left[h_{i-1},x_{t}\right]+b_{i}\right)\text{,} \end{array}$$

(3) $$\begin{array}{c} a_{t}=\tanh\;\left(W_{c}\cdot \left[h_{t-1},x_{t}\right]+b_{c}\right)\text{,} \end{array}$$

(4) $$\begin{array}{c} C_{t}=f_{t}\cdot C_{t-1}+i_{t}\cdot a_{t}\text{,} \end{array}$$

(5) $$\begin{array}{c} o_{t}=\sigma \left(W_{o}\cdot \left[h_{i-1},x_{t}\right]+b_{o}\right)\text{,} \end{array}$$

(6) $$\begin{array}{c} h_{t}=o_{t}\cdot \;\tanh\left(C_{t}\right)\text{,} \end{array}$$

where *w* denotes the weight and *b* denotes the bias amount of each gating unit.

### 2.2. Particle Swarm Optimization

PSO can be used to find the optimal solution quickly through the information interaction between particles. The particles in the algorithm are moving simultaneously, and all particles will generate memory and experience in the process of motion. Any individual particle will compare its experience with the experience provided by other particles in the process of finding the optimal solution, so that it will constantly be in the optimal solution. The PSO's velocity position update equation is shown in equation (7):

(7) $$\begin{array}{c} \left\{\begin{array}{c} v_{i,j}^{t+1}=\omega v_{i,j}^{t}+c_{1}r_{1}\left({pbest}_{i,j}^{t}-x_{i,j}^{t}\right)+c_{2}r_{2}\left({gbest}_{j}^{t}-x_{i,j}^{t}\right)\text{,}\\ x_{i,j}^{t+1}=x_{i,j}^{t}+v_{i,j}^{t+1}\text{,} \end{array}\right. \end{array}$$

where *w* is the inertia weight; *c*_1_ and *c*_2_ is the learning factor; *r*_1_ and *r*_2_ is the random number between [0,1]; *v*, *x*, *p*best, *g*best are the velocity component, position component, individual optimum, and population global optimum of the *i*th particle in the *j*th dimension at the *t*th iteration, respectively.

### 2.3. PSO-LSTM

#### 2.3.1. Flow of PSO Optimize Network Parameters

**Figure 2:**
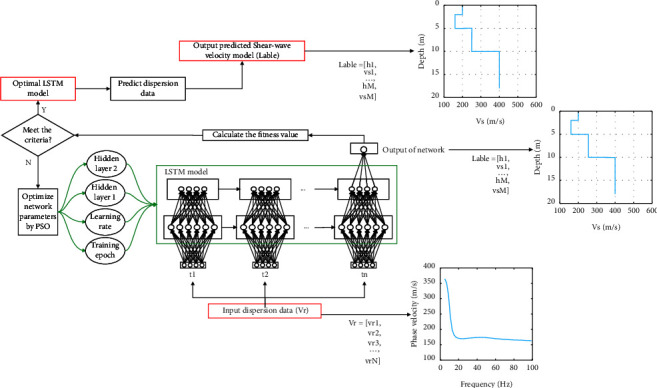
The flowchart of the proposed PSO-LSTM.

The selection of network parameters for LSTM is usually based on researchers' experience, and the low prediction accuracy of the model caused by artificial selection can be avoided if PSO is used to determine the parameters. The process of PSO to find the optimal network parameters is as follows: the number of hidden layer neurons, learning rate, and number of training rounds of key model parameters in LSTM are used as optimization-seeking variables for particles in different dimensions, and the optimal model parameters are obtained by continuously updating the velocity and position of particles and calculating and comparing the objective function fitness values so as to achieve the global optimum. The PSO-LSTM flow chart is shown in Figure 2. The PSO-LSTM flow is described as follows:

  Step 1 The data are divided into training and test sets in a 4 : 1 ratio. The input data is only the Rayleigh wave phase velocity; therefore, no normalization of the data is performed.
  Step 2 The number of hidden neurons, learning rate, and number of training rounds of the LSTM are set as the search parameters to initialize the population, and the search ranges of hidden neurons, learning rate, and number of training rounds are 50–300, 0.05–0.3, and 200–1000, respectively (the search ranges for these parameters are given based on the researcher's experience).
  Step 3 The prediction error of the PSO optimized LSTM is the fitness value of the particle, and the fitness value changes with the number of iterations, and the individual particle updates the individual optimal position and the global optimal position according to the fitness value and then updates its own speed and position according to equation (7).
  Step 4 Stop the iterative update when the fitness value of the particle stabilizes and determine the values of the number of hidden layer units, learning rate, and the number of training rounds.
  Step 5 Input the optimal parameters into the LSTM for training and prediction.

#### 2.3.2. Evaluation Metrics for PSO-Optimized LSTM Network Parameters

In the process of parameter optimization by PSO, PSO continuously updates the number of hidden layer units, learning rate, and training rounds to build LSTM models with optimized parameters for training and prediction. The particle fitness value is represented by the mean absolute percentage error (MAPE) of the prediction of shear-wave profiles on the test set. The lower the value of MAPE, the better the parameters found in this iteration, and the optimal parameters are determined. The MAPE is calculated as

(8) $$\begin{array}{c} MAPE=\frac{1}{N}\overset{N}{\underset{i=1}{\sum }}\left|\frac{y_{i}-\overset{\wedge }{y_{i}}}{y_{i}}\right|\text{,} \end{array}$$

where *N* is the sample size, *y*_*i*_ denotes the true value, and ${\overset{\wedge }{y}}_{i}$ denotes the predicted value.

The model is built with optimal parameters and trained after the optimization search is completed. The accuracy of the LSTM solution is tested by the mean squared error (MSE) between the predicted and true values. The lower value of MSE indicates the higher accuracy of the model solution, and the formula for calculating MSE is

(9) $$\begin{array}{c} MSE=\frac{1}{N}\overset{N}{\underset{i=1}{\sum }}{\left(y_{i}-\overset{\wedge }{y_{i}}\right)}^{2}\text{,} \end{array}$$

where *N* is the sample size, *y*_*i*_ denotes the true value, and ${\overset{\wedge }{y}}_{i}$ denotes the predicted value.

## 3. Synthetic Data Tests

The significant influence on the variation of Rayleigh wave dispersion curves characteristics is shear-wave velocity and thickness of the stratum [5], and the remaining parameters have a minor effect on it. In order to reduce calculation cost, only the shear-wave velocity and thickness are inverted. For the network training data, the scalar transfer algorithm is used to generate the data. The frequency band range of the dispersion curves is 5–100 Hz with a frequency interval of 3. The sample data were randomly generated within the upper and lower 50% of the theoretical model parameters [22]. The frequencies in the data are all the same, and to reduce the time cost, only the Rayleigh wave phase velocity in the dispersion data is used as the input data of the network model, and the stratum layer thickness and shear-wave velocity in the model parameters are used as the label data. In the article, the inertia weights, learning factors *c*1, and *c*2 of PSO are 0.8, 2.0, and 2.0, respectively. In the inversion of dispersion curves, the number of particles and iterations of PSO are 20 and 50, respectively. In PSO-LSTM, the number of particles and iterations of PSO are 10 and 20, respectively. All tests in the article were performed in the same environment. The software platform is PyCharm, and the programming environment is the Python language using the PyTorch framework. PyTorch and Python version are 1.10.2 and 3.6.13, respectively. The CPU and GPU of the computer used in this article are Intel Core i5-10400F and NVIDIA GeForce RTX 3060, respectively.

The objective function of the PSO to invert dispersion curve is

(10) $$\begin{array}{c} F=\sqrt{\left\{\frac{\overset{M}{\underset{i=1}{\sum }}{\left(V_{R}^{obs}\left(i\right)-V_{R}^{cal}\left(i\right)\right)}^{2}}{M}\right\}}\text{,} \end{array}$$

where *V*_*R*_^*obs*^ is the measured phase velocity of the Rayleigh wave; *V*_*R*_^*cal*^ is the theoretical phase velocity of the Rayleigh wave; and *M* is the number of points of frequency.

**Figure 3:**
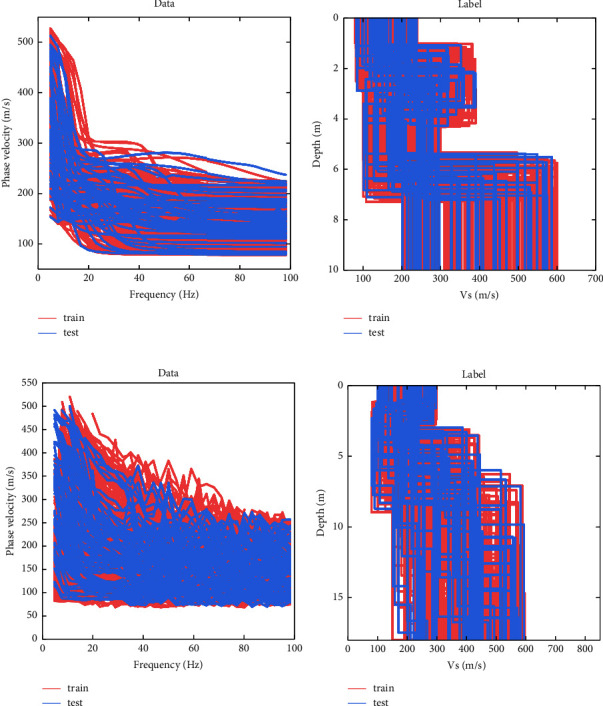
Sample data and labels for Models *A* and *B*. (a, c) Sample data of Model *A* and Model *B*. (b, d) Mean labels for Model *A* and Model *B*.

To verify the performance of PSO-LSTM, two typical geological models were designed. Model *A* is a four-layer geological model with increasing velocity with depth; Model *B* is a four-layer model with a low-velocity layer in the middle of the model; and the model parameters are shown in Table 2. Model *A* is tested without noise, and the data contain the dispersion data of the fundamental mode; Model *B* is tested with noise, and the data contain the dispersion data of the fundamental mode and second mode. The sample data of model *A* and *B* are shown in Figure 3. The sample data of model *A* contain no noise, and the sample data of model *B* have 10% Gaussian noise added to it.

### 3.1. Noiseless Synthetic Data Test

**Figure 4:**
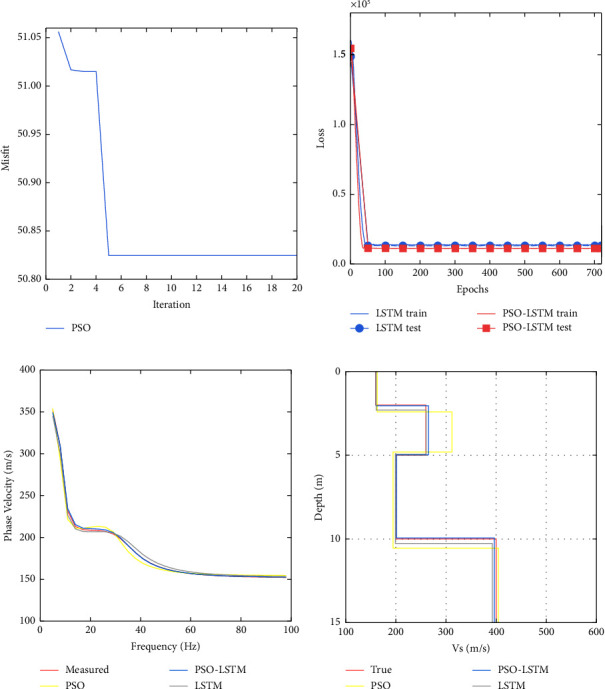
Training process of Model *A* and its prediction results: (a) convergence process of PSO; (b) network training error; (c) inverted dispersion curve; and (d) inverted shear-wave velocity profile.

To compare the inversion performance of PSO, LSTM, and PSO-LSTM, we tested these three inversion methods using Model A. The number of neurons in the hidden layer 1 and hidden layer 2 in the parameters of the LSTM network without PSO optimization given by experience are 160 and 118, respectively; the learning rate and the number of training rounds of the LSTM network are 0.17 and 719, respectively. The optimal LSTM network parameters by PSO search are as follows: the number of neurons in hidden layers 1 and 2 is 254 and 276, respectively; the learning rate is 0.0890; and the number of training rounds is 719. From Figures 4(a) and 4(b), we can see that using the model parameters searched in 20 iterations to train the network can already get reasonable training results. The same trend of training error and validation error in Figure 4(b) indicates that the model is well trained. From Figure 4(c), it can be seen that PSO, LSTM, and PSO-LSTM inverted dispersion curves fit well with the observed dispersion curves, indicating that they have found the optimal solution. In Figure 4(d), the shear-wave velocity profile of PSO inverted has a large deviation from the shear-wave velocity profile of real model in the second and fourth layers; the shear-wave velocity profile of LSTM inverted agrees better with the shear-wave velocity profile of real model, but there are also some deviations in the layer thicknesses of the second and fourth layers; our proposed PSO-LSTM works the best, and the inverse transverse velocity profile agrees almost perfectly with the shear-wave velocity profile of real model. The fit of the inverted model to the real model shows that the LSTM network is more suitable for inversion of dispersion curves than PSO; the prediction effect of LSTM network is better after PSO optimizes the network parameters of LSTM, and PSO optimizes the network parameters to play a role in improving the network accuracy.

### 3.2. Noisy Synthetic Data Test

**Figure 5:**
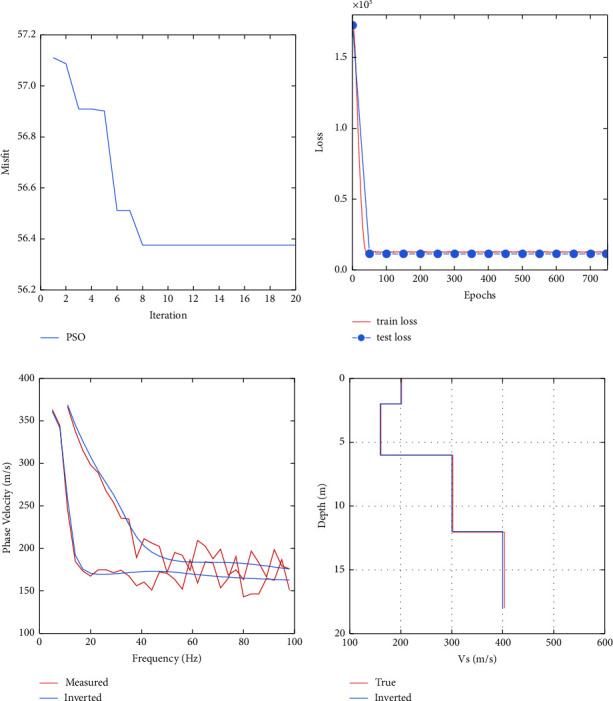
Training process of model *B* and its prediction results: (a) convergence process of PSO; (b) network training error; (c) inverted dispersion curve; and (d) inverted shear-wave velocity profile.

Noise is inevitable when acquiring real seismic data. The noise will make the extracted phase velocity deviate from the true value, making the inversion more difficult and reducing the accuracy of inverse results [10]. Therefore, the capability of PSO-LSTM to invert noisy data is necessary to be examined. In order to simulate the real data, 10% Gaussian noise is added to both Model *B* and the sample data of Model *B*. Then, the trained network is used to invert model *B*. The optimal LSTM network parameters are searched by PSO: the number of neurons in hidden layers 1 and 2, the learning rate, and the number of training rounds are 61, 239, 0.1140, and 747, respectively. From Figure 5(a), we can see that after 20 iterations, the function values have converged.  Figure 5(b) indicates that the model is well trained. In Figure 5(c), the dispersion curves fit well, and there is no significant deviation between the inverted dispersion curves and the observed dispersion curves.  Figure 5(d) shows that the PSO-LSTM can still reconstruct the shear-wave velocity model well after noise is added to the model. The reconstructed shear-wave velocity model fits well with the shear-wave velocity model of the real model, indicating that the inversion works well.

### 3.3. Stability Analysis

**Figure 6:**
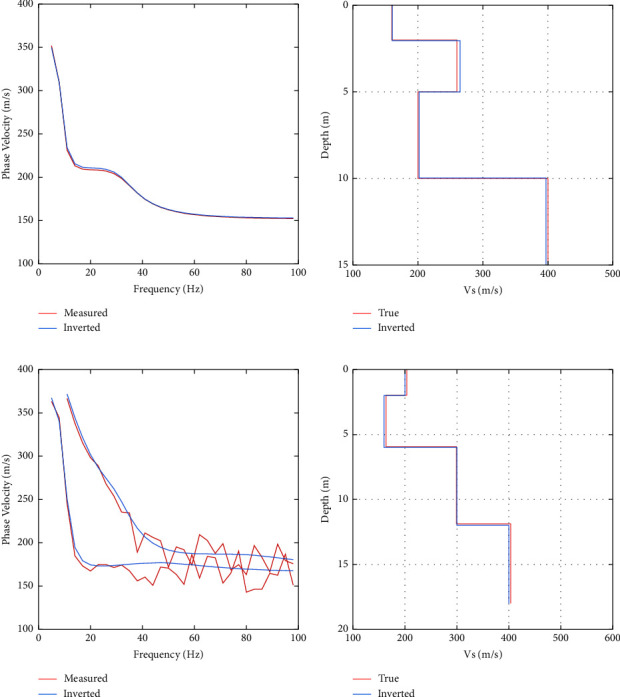
The inverse mean values of Model *A* and Model *B*. (a, c) Inverted dispersion curve of Model *A* and Model *B*. (b, d) Mean inverted shear-wave velocity profile of Model *A* and Model *B*.

To further evaluate the performance of the PSO-LSTM to invert the dispersion curves, the theoretical model test was repeated 10 times, respectively, and the mean and standard deviation of the inverse results were calculated. The mean and standard deviation are generally used to reflect the stability of the inverse results, the smaller the mean and standard deviation, the more stable the inverse results [23, 24]. Keeping the network parameters constant, the network is trained 10 times and predicted separately. The inverted results are shown in Figure 6 and Table 3. In addition, 10 inversion tests were also performed separately for PSO and LSTM on Model *A*, and the inverted results are added in Table 3. In model *A*, the maximum relative errors in the inverse results of PSO, LSTM, and PSO-LSTM are 20.76%, 5.85%, and 2.05%, respectively. The maximum standard deviations of the inverse results of PSO, LSTM, and PSO-LSTM in Model *A* are 57.37, 1.97, and 1.23, respectively. These results show that among PSO, LSTM, and PSO-LSTM, PSO-LSTM performs the best and is the most stable.

In Table 3, the maximum relative errors and maximum standard deviations of model *B* are 2.09% and 3.87. In Figure 6(c), the inverted dispersion curves are close to the real dispersion curves. From Figure 6(d), it can be seen that the reconstructed shear-wave velocity model and the real shear-wave velocity profile are well fitted. These show that PSO-LSTM is stable, and the inverted models from PSO-LSTM can accurately predict the real ones.

## 4. Real Data Test

**Figure 7:**
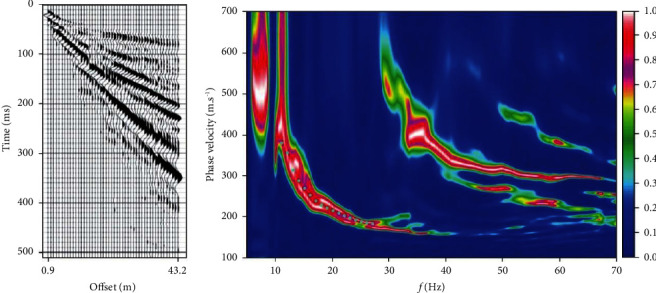
Wyoming: seismic record (a) and dispersion image (b) [25].

The next step will be to test the ability of PSO-LSTM to invert the actual data acquired from the Wyoming area of the United States [25]. The original seismic record is shown in Figure 7(a). Forty-eight 8 Hz vertical component geophones were used to collect data, the interval was 0.9 m, the minimum offset distance was 0.9 m, and the shock source was a hammer shock source.  Figure 7(b) shows the dispersion image extracted from the seismic record. The inverse test was performed using the dispersion curves picked up on the fundamental mode (solid dots in Figure 7(b)). The exploration depth was divided into five layers based on the logging data, and the set stratigraphic physical parameters are shown in Table 4.

**Figure 8:**
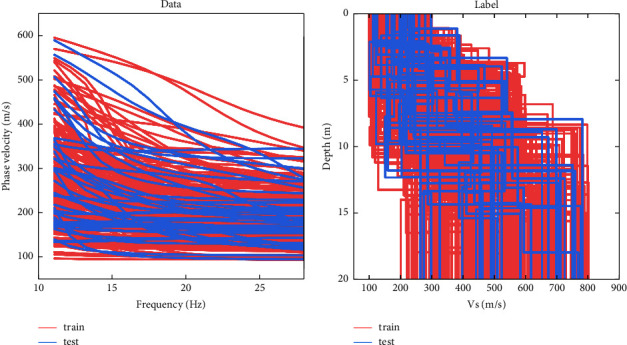
Sample data and labels for real data: (a) sample data of actual data and (b) labels of actual data.

**Figure 9:**
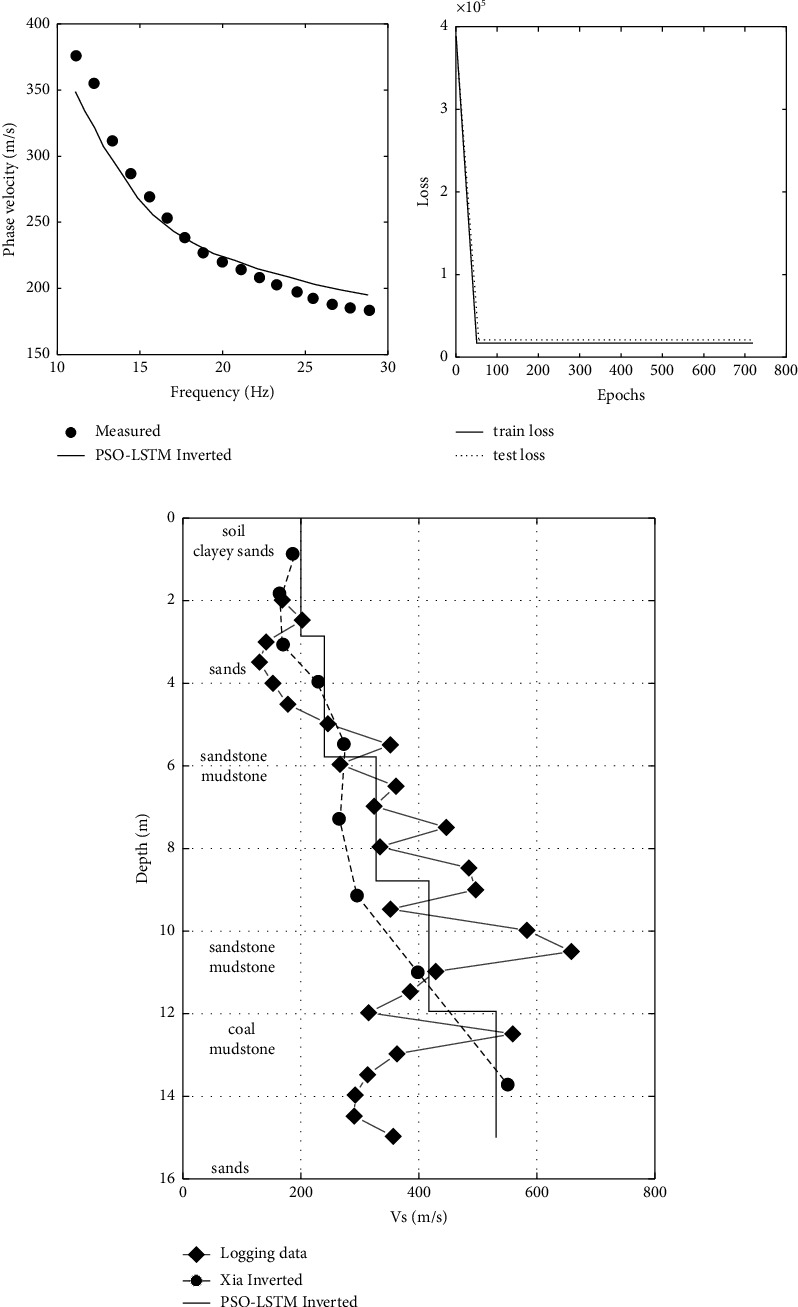
Inverted results of the measured data in Wyoming: (a) measured data and inverted dispersion curve; (b) network training error; and (c) inverted shear-wave velocity model.

250 sample data were created according to Table 4 using the fast scalar method, and the data are shown in Figure 8. The parameters of the PSO-LSTM obtained by PSO search are as follows: the number of neurons in hidden layers 1 and 2 are 217 and 192, respectively, the learning rate is 0.1760, and the number of training rounds is 719. Figures 9(a) and 9(c) show the inverse results, and Figure 9(b) shows the network training error. From Figure 9(a), it can be seen that the dispersion curve obtained from the inversion matches well with the measured curve. In Figure 9(c), the shear-wave velocity model obtained from inversion matches well with the logging data. The inverted shear wave velocity model is not only close to the logging data in terms of speed but also roughly consistent with the depth of the real formation. This shows that the results of PSO-LSTM inverted the actual data are reliable.

## 5. Conclusion

In this paper, we propose a dispersion curve inversion method based on a deep learning model. The method can avoid the manual parameter selection and improve the prediction accuracy of the network. In the specific test work, the optimal network parameters are first selected using PSO, and the prediction of the dispersion data is performed after training the model using the selected network parameters. PSO-LSTM achieved favourable inverse results in the model test. The maximum relative error and maximum standard deviation of PSO-LSTM in Model *A* and Model *B* are 2.05%, 2.09%, and 1.23, 3.87, respectively. The inverse results of Model *A* show that the PSO-LSTM can be successfully applied to the study of inversion of dispersion curves. The test results of Model *B* confirm the stability of PSO-LSTM. Even if the dispersion curve data contain some noise, the inversion results of PSO-LSTM are still reliable. Finally, the ability of PSO-LSTM to invert actual data is tested. The real data tests show that PSO-LSTM is practical and the inverse results are reliable.

## Figures and Tables

**Table 1:** Dispersion curves inversion studies carried out in recent decades (performed algorithms with their merits and demerits).

Algorithm	Reference number	Merits	Demerits	Compared with
Least-squares algorithm	[4]	(1) High calculation speed; (2) High precision of solution	(1) The appropriate initial model needs to be given; (2) The partial derivative needs to be calculated; (3) Easy to fall into local minima	None

Levenberg–Marquardt algorithm combined with the singular value decomposition technique	[5]	(1) High calculation speed; (2) Excellent stability	(1) The appropriate initial model needs to be given; (2) The partial derivative needs to be calculated; (3) Easy to fall into local minima	None

Occam algorithm	[6]	(1) High calculation speed; (2) High precision of solution; (3) Excellent stability	(1) The appropriate initial model needs to be given; (2) The partial derivative needs to be calculated; (3) Easy to fall into local minima	None

Genetic algorithm	[7]	(1) Excellent ability to escape from local minima; (2) Independent of selecting the initial model; (3) Calculation of partial derivatives is avoided	(1) Huge computational time cost; (2) Low accuracy of calculation	None

Genetic algorithm combining elite selection and dynamic mutation strategy	[8]	(1) Excellent stability; (2) Excellent ability to escape from local minima; (3) Independent of selecting the initial model; (4) Calculation of partial derivatives is avoided	(1) Huge computational time cost; (2) Low accuracy of calculation	Marquardt algorithm

Genetic algorithms combining marginal posterior probability density estimation	[9]	(1) Excellent ability to escape from local minima; (2) Independent of selecting the initial model; (3) Calculation of partial derivatives is avoided	(1) Huge computational time cost; (2) Low accuracy of calculation	None

Heat-bath simulated annealing algorithm	[10]	(1) Excellent ability to escape from local minima; (2) Independent of selecting the initial model; (3) Calculation of partial derivatives is avoided; (4) Suitable for parallel programming	(1) Low accuracy of calculation	Levenberg–Marquardt algorithm and fast simulated annealing algorithm

Artificial neural network	[18]	(1) Excellent stability; (2) High inversion efficiency	(1) Requires large amounts of training data; (2) Training the network costs a lot of time	Monte Carlo approach and gray wolf optimizer

LSTM based on the first height last velocity	[19]	(1) Excellent stability; (2) High inversion efficiency	(1) Requires large amounts of training data; (2) Training the network costs a lot of time	None

**Table 2:** Model parameters and search range (Vp, Vs, *h*, *ρ* represent *P*-wave velocity, *S*-wave velocity, stratigraphic layer thickness, and stratigraphic density, respectively).

Model	Layers	Parameters				Search range	
		Vs	Vp	*ρ*	*h*	Vs	*h*
		(m/s)	(m/s)	(g/cm^3^)	(m)	(m/s)	(m)
Model *A*	1	160	531	2.0	2	80∼240	1∼3
	2	260	862	2.0	3	130∼390	1.5∼4.5
	3	200	663	2.0	5	100∼300	2.5∼7.5
	4	400	1327	2.0	∞	200∼600	∞

Model *B*	1	200	663	1.9	2	100∼300	1∼3
	2	160	673	1.9	4	80∼240	2∼6
	3	300	1102	1.9	6	150∼450	3∼9
	4	400	1470	1.9	∞	200∼600	∞

**Table 3:** Mean values of Model *A* and *B* inverse results (IMV denotes the inverse mean value, RE denotes the relative error, and SD denotes the standard deviation).

Model *A*								
Parameters	True	PSO				LSTM		
		IMV		RE (%)	SD	IMV	RE (%)	SD
Vs_1_ (m/s)	160	162.81		20.76	6.27	156.84	1.98	0.81
Vs_2_ (m/s)	260	311.80		20.24	57.37	264.86	1.87	1.64
Vs_3_ (m/s)	200	194.58		14.76	45.42	195.79	2.11	0.96
Vs_4_ (m/s)	400	404.44		1.76	10.36	398.89	0.27	1.97
H_1_ (m)	2	2.42		19.92	0.53	1.88	5.85	0.36
H_2_ (m)	3	2.39		2.71	1.02	2.95	1.63	0.17
H_3_ (m)	5	5.74		1.11	2.18	4.91	1.73	0.41

*Model A*								
*PSO-LSTM*								
*Parameters*	*True*		*IMV*		*RE (%)*		*SD*	
Vs_1_ (m/s)	160		160.80		0.51		0.39	
Vs_2_ (m/s)	260		264.96		1.91		0.59	
Vs_3_ (m/s)	200		201.79		0.90		0.58	
Vs_4_ (m/s)	400		397.10		0.73		1.23	
H_1_ (m)	2		2.04		2.05		0.01	
H_2_ (m)	3		2.97		1.10		0.02	
H_3_ (m)	5		4.97		0.57		0.02	

*Model B*								
Vs_1_ (m/s)	200		203.74		1.87		0.93	
Vs_2_ (m/s)	160		163.66		2.09		1.24	
Vs_3_ (m/s)	300		299.09		0.30		1.66	
Vs_4_ (m/s)	400		403.64		0.91		3.87	
H_1_ (m)	2		1.98		1.22		0.02	
H_2_ (m)	4		3.96		1.05		0.04	
H_3_ (m)	6		5.94		0.93		0.06	

**Table 4:** Wyoming: search range and model parameter settings for inversion of PSO-LSTM (*σ* stands for Poisson's ratio) [26].

Layers	Vs	*h*	*σ*	*ρ*
	(m/s)	(m)		(g/cm^3^)
1	100∼300	1∼5	0.38	2.0
2	100∼400	1∼5	0.38	2.0
3	100∼600	1∼5	0.35	2.0
4	200∼600	1∼5	0.35	2.0
5	200∼800	∞	0.30	2.0

## Data Availability

The experimental data used to support the findings of this study are available from the corresponding author upon request.
